# Prevalence, correlates and help-seeking behaviour for depressive symptoms in rural Uganda: a population-based survey

**DOI:** 10.1017/gmh.2019.25

**Published:** 2019-10-21

**Authors:** J. Ssebunnya, G. Medhin, S. Kangere, F. Kigozi, J. Nakku, C. Lund

**Affiliations:** 1 Makerere University College of Health Sciences, Kampala, Uganda; 2 Aklilu Lemma Institute of Pathobiology, Addis Ababa University, Addis Ababa, Ethiopia; 3 Butabika National Mental Hospital, Kampala, Uganda; 4 Department of Psychiatry and Mental Health, Alan J Flisher Centre for Public Mental Health, University of Cape Town, Rondebosch, South Africa; 5 Health Service and Population Research Department, Centre for Global Mental Health, Institute of Psychiatry, Psychology and Neuroscience, King's College London, UK

**Keywords:** Community, depression, help-seeking, PHQ-9

## Abstract

**Background.:**

Depression is a common disorder characterized by delayed help-seeking, often remaining undetected and untreated.

**Objectives.:**

We sought to estimate the proportion of adults in Kamuli District with depressive symptoms and to assess their help-seeking behaviour.

**Methods.:**

This was a population-based cross-sectional study conducted in a rural district in Uganda. Sampling of study participants was done using the probability proportional to size method. Screening for depression was done using Patient Health Questionnaire (PHQ-9). The participants who screened positive also reported on whether and where they had sought treatment. Data collected using PHQ-9 was used both as a symptom-based description of depression and algorithm diagnosis of major depression. All data analysis was done using STATA version 13.

**Results.:**

With a cut-off score of ⩾10, 6.4% screened positive for current depressive symptoms and 23.6% reported experiencing depressive symptoms in the past 12 months. The majority of individuals who screened positive for current depression (75.6%) were females. In a crude analysis, people with lower education, middle age and low socio-economic status were more likely to have depressive symptoms. Help-seeking was low, with only 18.9% of the individuals who screened positive for current depression having sought treatment from a health worker.

**Conclusion.:**

Depressive symptoms are common in the study district with low levels of help-seeking practices. People with lower levels of education, low socio-economic status and those in middle age are more likely to be affected by these symptoms. Most persons with current depression had past history of depressive symptoms.

## Background

A growing body of evidence suggests that mental health problems are a major contributor to the burden of disease globally. Studies have shown that these disorders pose a growing challenge for health systems in developed and developing regions of the world (Whiteford *et al*. 2013). Mental disorders are estimated to contribute about 14% to the global disease burden; with depression, in particular, contributing 3.8% of the Disability Adjusted Life Years globally (Prince *et al*. 2007; Brhlikova *et al*. 2011; Ferrari *et al*. 2013).

Depressive disorders are a common form of mental disorders, occurring predominantly in adulthood and across all world regions (Charlson *et al*. 2013; Ferrari *et al*. 2013). The 2010 global burden of disease study identified depressive disorders as the second leading cause of disability globally; emphasizing the importance of including depressive disorders as a public health priority and implementing interventions to reduce its burden (Ferrari *et al*. 2010).

There is mounting evidence that clinical depression takes a serious toll on physical health and gives rise to considerable adverse effects on activities of daily living for extended periods of time, resulting in social isolation as well as comprising a source of significant economic burden (Greenberg *et al*. 2015). Several studies in African countries have documented the prevalence of depressive disorder ranging from 1.8% to as high as 42.4% among people with other physical conditions such as HIV/AIDS, heart disease and diabetes (Adewuya *et al*. 2006; Mbakwem & Aina, 2008; Agbir *et al*. 2010). The point prevalence rates of depression in the general population in Africa and the Middle East have been estimated between 1.1 and 29.3% (Travers *et al*. 2013).

In Uganda, the few available prevalence studies have estimated varying point prevalence rates of depression in the general population, of up to 29.3% (Bolton *et al*. 2004; Ovuga *et al*. 2005a, b; Kinyanda *et al*. 2011). Furthermore, studies found the female gender to be independently associated with an increased risk of depressive disorder (Kinyanda *et al*. 2011). Like in many other parts of the world, the major difficulty with depressive disorder in Uganda is its conceptualization and recognition; and thus, many cases remain undetected and therefore untreated (Okello & Neema, 2007).

Little is known about the prevalence of depression, its associated factors as well as help-seeking among affected persons in general populations in rural Uganda. In this paper, we present findings from a population-based survey of adults in a rural district in Uganda, examining the prevalence and correlates of depressive symptoms, stigmatizing beliefs and the help-seeking behaviours.

## Methods

The outcome data reported were collected in a cross-sectional population-based survey of adults aged 18 years and above, using a standardized screening tool for depressive symptoms, the Patient Health Questionnaire-9th version (PHQ-9) (Kroenke *et al*. 2001).

### Study setting

This study was conducted in Kamuli district, a predominantly rural district located in Eastern Uganda, 140 km away from the capital city. Administratively, the district is made up of two counties, 10 sub-counties, 79 parishes and 755 zones/villages. For health service delivery, it is divided into two health sub-districts, each having a number of health facilities at various levels. The district has a total fertility rate well above the national average (6.8 and 5.8, respectively), and was estimated to have a population of 500800 at the time of the survey; with the males constituting 48.1% and the females 51.9% (UBOS, 2016). The population was predominantly young, with an estimated 59% being children below 18 years of age (Uganda Bureau of Standards UBOS, 2011).

The district has a fair distribution of public health facilities, including one general hospital and 34 lower level health centres [Health Centre (HC) II, III and IV]. HC IIs are found at the parish level. These are the first level of interaction between the formal health sector and the communities, and provide out-patient care (especially for the common diseases such as malaria) as well as community outreach services. A HC II is headed by an enrolled nurse, working with a midwife and nursing assistants. HC IIIs are found at sub-county level. These provide basic preventive and curative care as well as support supervision of the community and HC IIs under them. There are also provisions for laboratory services for diagnosis and maternity care. HC IIIs are headed by a medical clinical officer, working with a number of nurses and midwives. HC IVs are at county/health sub-district level. In addition to the services found at HC III, HC IVs also provide in-patient services and are equipped with theatres for carrying out appropriate emergency operations at that level. In some districts, they serve as district referral hospitals. A HC IV is headed by a medical officer, working with several other various cadres of health workers.

With one psychiatric clinical officer and a psychiatric nurse in place, mental health care in the district was available at the general hospital and some of the lower-level health facilities as an integral component of primary health care, although estimates of the number of people with MNS disorders accessing care was not available (Hanlon *et al*. 2014). According to its socio-economic and health indicators, the district is typically representative of the majority rural districts in Uganda.

### Sampling design

This baseline survey was conducted in 30 villages within Kamuli district, prior to the implementation of the mental health care plan (Kigozi *et al*. 2016); conducted as part of the PRogramme for Improving Mental health carE (PRIME) study (Lund *et al*. 2012). According to the hierarchical administrative structure in Uganda, a village is the smallest unit and comprises 30–500 households, depending on the location. Several villages are combined to make a parish, and this comprises several hundred or thousands of households. A number of parishes then constitute a sub-county, and a couple of sub-counties form a county. A district may have one or more counties.

A list of all parishes within the district was obtained including the total number of households within each parish. Applying probability proportional to size sampling method, 30 villages were randomly selected, from 30 parishes. Although largely a rural district, two out of the 30 villages were relatively urban (within Kamuli Town Council), another 12 were peri-urban having relatively big trading centres, while the rest (16) were typically rural and remote villages.

While the target sample per village was 60, in some cases the actual sample size was as a low as 25 due to constraints on the length of time field workers could remain in that village during working hours. More details on the sampling have been described in a paper by Rathod *et al*. (2016). The sampling procedure gave a total of 1290 participants completing the survey (one participant from each household). This sample size was considered adequate to provide an estimate for changes in treatment coverage for depression as a result of implementing a district mental healthcare plan, as part of PRIME (Lund *et al*. 2012). Changes in treatment coverage were to be evaluated through a follow-up community-based survey in the same district (De Silva *et al*. 2016).

### Questionnaire design and measurements

The questionnaire consisted of two parts. Part 1 covered: basic socio-demographic characteristics; a screening tool for alcohol use disorders, the Alcohol Use Disorders Identification Test (AUDIT) (Babor *et al*. 2001); items assessing treatment-seeking and internalized stigma for problems with alcohol; screening tool for depression; the PHQ-9 (Kroenke *et al*. 2001); items assessing treatment-seeking and internalized stigma for depression as well as suicidality. Part 2 consisted of sections pertaining to detailed socio-demographic characteristics and household asset index (specific questions relating to household property/assets); mental health knowledge, attitudes and behaviour; health care usage and out-of-pocket expenditures; and disability severity. The questionnaire was developed with input from all the five participating country research sites and international partners.

Depressive symptoms were assessed using the 9-item PHQ-9, developed based on the Diagnostic and Statistical Manual of mental disorders to assess depression in the primary care setting. This instrument asks the respondent to indicate the frequency of various symptoms over the past 2 weeks. A standard cut-off score of ⩾10 was used for this study. However, in light of the low detection rate, the tool was subsequently validated in the Kamuli site in Uganda, during which a potential cut-off score of ⩾5 was proposed (sensitivity 67.4 and specificity 78.1) although this would mean a significant trade-off between sensitivity and low positive predictive values (PPV). (Nakku *et al*. 2016). The scores are categorized into severity grades, typically as mild (score of 5–9), moderate (score of 10–14), moderately severe (score of 15–19) and severe (score of 20 and above). The reliability coefficient, Cronbach's alpha, for the PHQ-9 scale was 0.816

Recent depression was identified by asking respondents whether they had experienced persistent depressive symptoms or most of the already described PHQ-9 depressive symptoms for a period of at least 2 weeks in the past 12 months. Help-seeking for participants with depressive symptoms was assessed by asking for where the participants had sought help and the kind of help/treatment they had received. The sub-section on household asset index had 11 questions asking about the socio-economic characteristics of the home and availability of various household amenities, as a proxy variable for household assets. Food security was assessed by a single item asking whether any household member had gone hungry the previous month due to lack of resources.

The entire questionnaire was translated into Luganda which is a widely spoken local language, and then back-translated.

### Interview procedure

Data collection was undertaken by field workers, who were trained for 4 days on all aspects of conducting the interview, and the content of the instrument. This was followed by 2 days of pilot-testing and refinement of the tool. After consenting, Part1 of the questionnaire was administered to every study participant to provide basic socio-demographic information and to screen for depression and alcohol use disorder. All participants who screened positive on the screening tool, those reporting recent depression, plus a random sample of approximately 10% of those who screened negative proceeded to complete Part 2 of the questionnaire. Through the process, a total of 490 participants completed the full interview. Field data collection was supervised by a Field Supervisor and a Research Officer, who were both university graduates of Clinical Psychology.

### Data management and analysis

Data collection was done by administering the questionnaire programmed on a Tablet device (using Mobenzi Researcher technology). As the study site was a rural area with a limited internet connection, the field workers initially collected data with pencil and paper, administering the questionnaire in Luganda. A data entry operator then entered the paper questionnaire responses into an English-language version of the tablet questionnaire application. The application automatically transmitted completed questionnaire data via mobile network to a secured server housed by the application provider. The data were then automatically deleted from the device.

The analysis was done using Stata version 13 (Stata for Windows). Reporting on the prevalence of Alcohol Use Disorders was done using a cut-off score of ⩾8 on the AUDIT tool. Descriptive analyses were done, summarizing the socio-demographic characteristics of the study participants, as well as those who screened positive (score of ⩾10) on the PHQ-9. Symptom-based severity of probable depression was reported by classifying PHQ-9 score into none (score of 0–4), minimal depressive symptoms (5–9), mild depressive symptoms (10–14), moderately severe depressive symptoms (15–19) and severe depressive symptoms (20+). We also used algorithm of PHQ-9 to classify study participants into two groups and reported prevalence of major depression. Assessment of help-seeking and internalized stigma as well as assessment of the association between depressive symptoms and characteristics of study participants was done for those who scored 10 or more points on PHQ-9. Pearson χ^2^ was used to assess bivariate associations between (a) being PHQ positive (i.e. having a score of 10 or more) and background characteristics, (b) item-based stigma and help-seeking with recent and current probable depression among screen positives. Logistic regression was used to model the associations of help-seeking with demographic and economic status among study participants who screened positive. The strength of association was summarized using odds ratio with corresponding 95% confidence intervals and findings were reported as being statistically significant whenever *p* value was <0.05.

### Ethical considerations

The study was approved by the Uganda National Council of Science and Technology, and the School of Medicine Research Ethics Committee (SOM-REC) at Makerere University College of Health Sciences, together with the Human Research Ethics Committee of the Faculty of Health Sciences, University of Cape Town. Informed consent was obtained after providing potential participants with adequate information about the study, as well as the potential benefits and risks involved. Participants reporting depressive symptoms and suicidal ideation were referred to the Psychiatric unit at the general hospital.

## Results

### Prevalence of depressive symptoms

Half the number of study participants were aged 18–34 years, while 26.2% were aged 45 years and above, implying that most participants were youths. A bigger proportion of the participants (two thirds) were females. Furthermore, the majority of the participants (nearly three quarters) had not gone beyond primary school with their education; with a relatively big proportion (18.8%) not educated at all; as indicated in Table 1.

**Table 1.** Associations of background characteristics and screening positive for depressive symptoms in Kamuli district, Uganda

Background characteristics	Total respondents number (%)**	PHQ positive number (%)	*p* value
Total study participants			
Age			
⩽24	281 (21.8)	13 (4.6)	0.006
25–34	367 (28.5)	22 (6.0)	
35–44	302 (23.5)	12 (4.0)	
45+	337 (26.2)	34 (10.1)	
Sex			
Male	442 (34.3)	20 (4.5)	0.052
Female	848 (65.7)	62 (7.3)	
Education			
Less than primary school	243 (18.8)	26 (10.7)	0.001
Primary school	677 (52.5)	45 (6.7)	
Secondary and above	370 (28.7)	11 (3.0)	
Employment status			
Employed	631 (48.9)	36 (5.7)	0.451
Unemployed	467 (36.2)	35 (7.5)	
*Other*	192 (14.9)	11 (5.7)	
Sub-sample of study participants			
Marital status			
Single	42 (11.7)	16 (38.1)	0.007
Partnered	244 (68.2)	43 (17.6)	
Widow(er)	39 (10.9)	11 (28.2)	
Separated/Divorced	33 (9.2)	11 (33.3)	
Socio-economic status			
Low	104 (29.1)	33 (31.7)	0.023
Average	125 (34.9)	21 (16.8)	
High	129 (36.0)	27 (20.9)	

With a cut-off score of 10 for the PHQ-9 questionnaire, 82 out of the 1290 participants (6.4%) screened positive for current depression; while 304 (23.6%) reported having depressive symptoms for a period of at least 2 weeks, in the past 12 months. The majority of those who screened positive were persons aged 45 years and above; and mostly females. Most of them (over 86.6%) were persons of either primary level education or below. Only a smaller proportion (13.4%) had gone up to secondary school or beyond. However, going by the PHQ-9 categorical algorithm, 28 study participants had major depression, giving a prevalence of 2.2%.

Furthermore, most of those who screened positive for current depression (89%) also had a history of depressive symptoms in the past 12 months. Only nine out of the 82 participants that screened positive for current depression (11%) had not experienced depressive symptoms in the previous 12 months. However, many of the participants with history of depressive symptoms (77.3%) screened negative for current depression, implying that their depressive symptoms had resolved.

Similarly, there was a correlation between middle age and depression. Food insecurity was associated with depression, but this was only marginally significant. There was a significant association between depressive symptoms and low socio-economic status as well as marital status.

The overall prevalence of Alcohol Use Disorders was 1.7% and it was much higher (3.7%) among depressed persons than among non-depressed ones.

### Suicide ideation

A total of 70 participants with both current and past depressive symptoms were further assessed for suicide ideation. Six of them (8.6%) had suicidal thoughts and had actually made suicide plans. Three out of the six (50%) of them had attempted suicide. Similarly, 233 persons with only past depressive symptoms were asked for suicidal ideation, and 11 of them (4.7%) reported having had suicidal thoughts. Six of the 11 (54.5%) had made suicidal plans, and four of them had actually attempted suicide.

### Stigma and help-seeking

As indicated in Table 2, exceptionally high proportions of participants with depressive symptoms felt out of place, were disappointed in themselves, thought their life was ruined and felt shame. Compared to those with past depressive symptoms, persons with current depression were more likely to report feeling out of place, discriminated against, emotionally distant or that their life had been ruined.

**Table 2.** Internalized stigma and help-seeking behaviour for recent and current depressive symptoms

Indicators of internalized stigma and help-seeking	Recent probable depression total number = 303 *n*(%)	Current probable depression total number = 82 *n*(%)	*p* value
Internalized stigma			
Feel out of place	218 (72.0)	71 (86.6)	0.007
Shame	143 (47.2)	48 (58.5)	0.069
Disappointed in self	205 (67.7)	67 (81.7)	0.014
Life is ruined	211 (69.6)	70 (85.4)	0.004
Decision making hindered	150 (49.5)	42 (51.2)	0.785
Can't contribute to society	84 (27.7)	30 (36.6)	0.117
Discrimination	108 (35.6)	39 (47.6)	0.047
Feel patronized	107 (35.1)	38 (46.3)	0.063
Ignored	103 (34.0)	32 (39.0)	0.400
Distance	64 (21.1)	31 (37.8)	0.002
Ability to achieve undermined	140 (46.2)	47 (57.3)	0.074
Help-seeking^a^			
Spoken to anyone about problems	166 (54.8)	48 (58.5)	0.529
Spoke to friend/neighbour	43 (14.2)	8 (9.8)	0.297
Spoke to employer/co-worker	3 (1.0)	1 (1.2)	0.874
Spoke to spouse/partner	32 (10.6)	6 (7.3)	0.375
Spoke to other family member	60 (19.8)	18 (22.0)	0.660
Spoke to religious official	27 (8.9)	8 (9.8)	0.801
Spoke to health worker	31 (10.2)	14 (17.1)	0.084
Not spoken to anyone	5 (1.7)	2 (2.4)	0.676

a Spoken to anyone about problems.

Over half of the participants with current and recent depression had spoken to someone about their problems (58.5% and 54.6%, respectively). They had mostly spoken to family members, health workers, friends/neighbours and religious leaders.

### Correlates of help-seeking for depressive symptoms

A reasonably lower proportion of participants reported having received treatment for their problems (only 28% of those with current depressive symptoms and 24% of those with past depressive symptoms). The majority of these had mild depressive symptoms (current) (Table 3).

**Table 3.** Correlates of help-seeking among the study participants who screened positive

Characteristics	Individuals who screened positive (*N* = 82) *n*(%) *N* = 82	Screen positives who sought help (*N* = 48) *n*(%)	*p* value	Crude OR	95% CI for crude OR
Age					
⩽24	13 (16.1)	6 (46.2)	0.539	Reference	
25–34	22 (27.2)	15 (68.2)		2.5	0.61–10.26
35–44	12 (14.8)	6 (50.0)		1.17	0.24–5.62
45+	34 (42.0)	21 (61.8)		1.88	0.52–6.86
Sex					
Male	20 (24.4)	11 (55.0)	0.712	Reference	
Female	62 (75.6)	37 (59.7)		1.21	0.44–3.35
Education					
Less than primary	26 (31.7)	13 (50.0)	0.058	Reference	
Primary school	45 (54.9)	25 (55.6)		1.25	0.48–3.29
Secondary and above	11 (13.4)	10 (90.9)		10.00	1.11–89.77
Marital status					
Single	16 (19.8)	12 (75.0)	0.375	Reference	
Partnered	43 (53.1)	23 (53.5)		0.38	0.11–1.38
Widow(er)	11 (13.6)	7 (63.6)		0.58	0.11–3.10
Separated/divorced	11 (13.6)	5 (45.5)		0.28	0.05–1.43
Employment status					
Paid or self	36 (43.9)	20 (55.6)	0.149	Reference	
Unemployed	35 (42.7)	24 (68.6)		1.75	0.66–4.61
Other	11 (13.4)	4 (36.4)		0.46	0.11–1.84
Quintiles of asset-based wealth index				
Low wealth	37 (45.7)	19 (51.4)	0.210	Reference	
Middle wealth	27 (33.3)	15 (55.6)		1.18	0.44–3.21
Highest wealth	17 (21.0)	13 (76.5)		3.08	0.85–11.22
Household food security					
Food insecure	40 (49.4)	21 (52.5)	0.210	Reference	
Food secure	41 (50.6)	26 (63.4)		1.57	0.65–3.81

By proportion, more participants aged 45 years and above had sought help/treatment for their problems. However, there was no statistically significant difference in help-seeking in terms of age. Similarly, persons with at least a primary level of education were more likely to seek treatment for depressive symptoms as compared to those who had not gone to school. There was no significant difference in help-seeking by marital status or employment status. The socio-economic status of the participants that screened positive was further assessed using questions on food security and asset index; and found to have a significant effect on help-seeking, as the odds of seeking treatment were higher for persons from middle and higher wealth indices compared to those in low wealth index.

## Discussion

Using a standard cut-off score of ⩾10 for the PHQ-9, the results indicate that 6.4% of the participants screened positive for depression, while 23.6% reported having depressive symptoms for at least 2 weeks in the past 12 months. The majority (92%) of individuals who screened positive for current depression also reported having depressive symptoms in the past 12 months; implying that their depressive symptoms persisted for quite some time. The findings corroborate some earlier studies that have estimated the prevalence of depression in the general population in Africa to be 1.1–29.3% (Travers *et al*. 2013). For example, Ferrari *et al*. (2013) reported the average prevalence of depression in sub-Saharan Africa to be 5.5%. Although no broad study has been done to assess the prevalence of depression in the general population in Uganda, a few localized studies on the general population have reported varying prevalence rates for depression. In a survey of 600 homes in Masaka and Rakai districts in Uganda, 21% of the respondents (*n*  =  587) met the DSM-IV criteria for diagnosis of depression (Bolton *et al*. 2004). Similarly, Ovuga *et al*. (2005a, b) reported a prevalence rate of 17.4% in two districts (Adjumani and Bugiri). While the former was a study done in two HIV/AIDS-stricken districts, the latter was done in two contrasting districts with one being a post-conflict and less socially cohesive district; and the other being relatively stable. Some exceptionally higher rates (as high as 67%) have been reported in the war-affected Northern Uganda region (Ovuga *et al.* 2005b). The persistence of the depressive symptoms for a period of 12 months or longer in this rural community could be attributed to the fact that many of the affected persons were less likely to have their depressive symptoms detected, or access appropriate treatment at the health facilities. It could also be attributed to the poor recognition of depression, as non-disruptive symptoms are often ignored and depression generally not considered a health problem by lay people (Okello & Neema, 2007). It should be noted that earlier studies have shown varying ranges of the prevalence of depression partly because of the differences in the tools used as well as the contextual and demographic differences of the study populations.

The socio-demographic characteristics of the participants were representative of a typical rural setting in Uganda. The results further showed an imbalanced distribution of participants in terms of sex, with the majority being females; which is partly attributable to the fact that more females are likely to be found at home during day time than males. Statistical analysis further showed a significant relationship with age, as the majority of those who screened positive (41.4%) were adults aged 45 years and above. This could be explained by the fact that elderly persons tend to face a lot of challenges in life associated with responsibilities. Moreover, they are faced with numerous physical, psychological and social role changes that challenge their sense of self and capacity to live happily, as reported by Singh & Misra (2009). In a related study by Ovuga *et al*. (2005a) respondents aged 40 years and above were twice more likely to be moderately depressed than those younger. The finding is also in line with the 2004 and 2010 global burden of disease studies that point to an increase in the risk of non-communicable diseases with age. According to the 2004 GBD study, the disease burden for LAMICs was much higher (48%) among persons of age group 15–59 years. Similarly, the 2010 global burden of disease study reported the largest proportion of Years Lost due to Disability from depressive disorders to occur at working ages between 15 and 64 years, peaking in the 20s and gradually decreasing into the older ages (WHO, 2004; Ferrari *et al*. 2013).

The proportion of women that screened positive for depression was much higher than that of men (75.6% compared with 24.4%). This is in line with several other studies that have found the female gender to be independently associated with the risk of depressive illness (Ovuga *et al*. 2005a; Kinyanda *et al*. 2011). Similarly, the 2004 global burden of disease study puts the burden of depression at 50% higher among females than males (WHO, 2004).

Findings further showed that a significantly larger proportion of participants with lower education levels screened positive for depression than participants with higher education levels. It is known that lower levels of educational attainment are often linked to higher unemployment rates and consequently lower socio-economic status, which may explain this relationship (Lund *et al*. 2010). Similarly, more persons who screened positive for Alcohol Use Disorders also reported depressive symptoms, suggesting a relationship between alcohol use and depression. Co-occurring alcohol use and depression have been associated with greater severity, heightened suicide risk and prolonged symptom course (Danzo *et al*. 2017). However, in this study, there was inadequate data to report on the association of depression and severity of alcohol use.

A substantial proportion of the participants who screened positive (7.6% for current depression and 4.9% for past depression) had suicidal tendencies. Although the relationship was not statistically significant, it is clear that findings affirm a link between depression and suicidal ideation. The suicidal risk has been said to vary depending on the socio-demographic status and clinical presentation (Srivastava & Kumar, 2005; Izadinia *et al*. 2010). In a related study that compared two districts (Ovuga *et al*. 2005b), the district that had a high prevalence of depression also had a higher prevalence of suicide ideation, largely attributed to psychosocial distress.

It is clear from the findings that in this study area, seeking help for depressive symptoms is relatively uncommon, as a small proportion of those who screened positive had sought treatment for their problems. As indicated in the findings, male participants with lower levels of education and those in the low wealth index were less likely to seek help for their depressive symptoms. This seems to reflect a trend, observed elsewhere, that the wealthy have more access to treatment as they can afford it (including transport and healthcare costs); and may be more educated and aware of the availability of services. Several studies have indicated that financial costs associated with care tend to influence the help-seeking behaviour (Nsereko *et al*. 2011; Evans-Lacko *et al*. 2017); with the poor being less likely to seek mental health care. Similarly, the proportion of those with current depressive symptoms who had sought treatment was more than that of persons with past depressive symptoms, implying that there had been some improvement in the help-seeking behaviour over time. More respondents with mild depressive symptoms (current) reported having sought treatment as compared to those with moderate or severe depressive symptoms. We are aware that this finding runs contrary to what one might expect, and there are several possible explanations. For example, those who sought treatment may have subsequently seen their symptoms remit, or there may have been measurement error, given the small numbers who reported seeking care. It should further be noted that help-seeking among persons with depression is influenced by the conceptualization of the problem. For example in one local study by Okello and Neema (2007), depression was generally perceived as ‘an illness of thoughts' that does not require western medicine but rather culturally accepted corrective traditional therapies.

### Limitations

Several limitations of this study need to be acknowledged: first, the findings are based on the PHQ-9, which is a screening tool that can only detect probable depression. Some of the participants who screened positive therefore might not necessarily have had diagnosable clinical depression. The survey did not go beyond screening to conduct a thorough examination and investigate the possible reasons for depressive symptoms. Secondly, data collectors were trained on aspects of conducting the interview, but we did not establish their inter-rater reliability. Thirdly, the survey findings are exclusively based on participants’ responses and there was no provision for assessing the authenticity of their responses. Fourthly, this is a cross-sectional survey which allows us to report associations but limits the extent to which we can draw any causal attributions from the associations observed.

### Recommendations

Based on the findings we wish to make the following policy recommendations: that public education programmes need to be undertaken to raise mental health awareness in the communities and enhance help-eeking behaviour; that evidence-based mental health care needs to be integrated into primary health care settings in rural Uganda, to make services readily available and narrow the treatment gap observed in this study. We further recommend a longitudinal study to report on possible causal pathways in the relationships identified in this study and community-based surveys in this district to assess whether the implementation of the PRIME mental health care plan has led to a reduction in the treatment gap.

## Conclusion

Depressive symptoms are quite common in this general rural Ugandan population, especially among people of lower education levels and lower socio-economic status; and the majority live with these symptoms undetected and thus untreated. Public education programmes need to be undertaken to raise mental health awareness in the communities and enhance help-seeking behaviour; and service accessibility needs to be improved, for example by providing mental health care through primary care services.

## Authors’ contribution

Js participated in the design of the study, supervised the data collection and drafted the manuscript. SK and GM participated in the analysis of the data. DK and SN participated in the data collection and commented on the draft. JN, FK and CL conceived and designed the study. FK, JN, GM and CL revised the manuscript providing intellectual content. All authors commented on and approved the final manuscript.
